# Letter to the editor - “Re: Tinea labialis in a patient with Schnitzler syndrome on interleukin-1 receptor antagonist”

**DOI:** 10.1016/j.jdcr.2025.10.071

**Published:** 2025-11-19

**Authors:** Breethiga Velusamy, Thais Pincelli, Olayemi Sokumbi

**Affiliations:** a Department of Dermatology, Research Fellow, Mayo Clinic, Jacksonville, Florida; b Department of Dermatology, Associate Professor of Dermatology, Mayo Clinic, Jacksonville, Florida; c Department of Dermatology, Professor of Dermatology and Laboratory Medicine & Pathology, Mayo Clinic, Jacksonville, Florida

*To the Editor:* We read with great interest the recent case by Mooney, Nathan et al, describing “tinea labialis” attributed to *Curvularia* species in *JAAD Case Reports*. We commend the authors for reporting this unusual fungal infection of the lip. However, we would like to respectfully clarify the terminology used. The term “tinea” is specifically reserved for superficial fungal infections of keratinized tissues caused by dermatophytes, namely *Trichophyton, Microsporum*, and *Epidermophyton* species. These organisms possess the unique ability to digest keratin, producing the characteristic annular lesions with raised borders and central clearing on the skin. Infections of the hair and nails present differently, such as alopecia in tinea capitis or onychodystrophy in tinea unguium.^1^ In contrast, *Curvularia* is a nondermatophyte, dematiaceous mold. When it affects the skin or subcutaneous tissue, the appropriate classification is cutaneous phaeohyphomycosis, not ‘tinea’.^2^ A similar distinction applies to *Candida* infections: although *Candida* can cause superficial mucocutaneous disease, these presentations are described as candidiasis (eg, cutaneous candidiasis or candidal onychomycosis), not ‘tinea’. Thus, restricting the term “tinea” to dermatophyte infections preserves both clinical and microbiological accuracy. We, therefore, suggest that the presented case be more precisely described as a *Curvularia* infection of the lip, or more specifically, cutaneous phaeohyphomycosis rather than “tinea labialis”. This distinction is not only terminologically important but also helps guide clinicians to consider a broader differential diagnosis for pigmented fungal infections and to select the most appropriate antifungal therapy.

## Conflicts of interest

None disclosed.
